# Associations of TNF‐α Expression With Self‐Esteem in Autism Spectrum Disorder

**DOI:** 10.1002/npr2.70047

**Published:** 2025-10-08

**Authors:** Yoshinori Kayashima, Takahira Yamauchi, Tsutomu Takeda, Kazuki Okumura, Rio Ishida, Kazuhiko Yamamuro, Michihiro Toritsuka, Takashi Okada, Nakao Iwata, Manabu Makinodan

**Affiliations:** ^1^ Department of Psychiatry Nara Medical University School of Medicine Kashihara, Nara Japan; ^2^ Department of Psychiatry Fujita Health University School of Medicine Toyoake Aichi Japan; ^3^ Division of Transformative Psychiatry and Synergistic Research, International Center for Brain Science (ICBS) Fujita Health University School of Medicine Toyoake Aichi Japan

**Keywords:** autism spectrum disorder, contingencies of self‐worth, self‐esteem, the personal sense of power, tumor necrosis factor‐alpha

## Abstract

**Aim:**

This study aimed to explore the relationship between self‐esteem and tumor necrotic factor‐alpha (TNF‐α) expression in individuals with autism spectrum disorder (ASD). Self‐esteem was assessed using the Contingencies of Self‐Worth (CSW) scale, with a focus on external and internal contingencies, and TNF‐α expression was measured, given its association with both ASD pathophysiology and self‐esteem in prior studies.

**Methods:**

We enrolled 51 high‐functioning individuals with ASD and 34 typically developed (TD) individuals. Self‐esteem was assessed using the Japanese version of the CSW scale, which evaluates seven domains, and the Personal Sense of Power. TNF‐α expression in plasma was quantified via ELISA. Correlations of CSW scores and the Personal Sense of Power with TNF‐α levels were analyzed using multiple regression models adjusted for confounding factors such as age, sex, education level, and autistic symptoms.

**Results:**

In ASD individuals, TNF‐α expression was significantly negatively correlated with the external CSW domain of others' approval and showed a trend toward negative correlations with appearance and relationship harmony. These correlations were not observed in the TD individuals. Likewise, the Personal Sense of Power within family settings showed a trend toward positive correlations with TNF‐α expression in ASD individuals, but not in TD individuals.

**Discussion:**

This study highlights the implication of TNF‐α levels in the self‐esteem of ASD individuals, particularly in interpersonal relationships. Lower TNF‐α expression was associated with higher self‐esteem in social contexts, independent of the severity of autistic symptoms. These findings suggest a biological link between inflammatory pathways and self‐esteem in ASD, contributing to a deeper understanding of the interplay between immune function and psychological well‐being in this population.

## Abstract 2

In adults with autism spectrum disorder (ASD), higher the pro‐inflammatory cytokine TNF‐α levels were significantly associated with lower self‐worth based on others' approval. These findings highlight the biological significance of how individuals with ASD perceive their interpersonal relationships.

## Graphical abstract

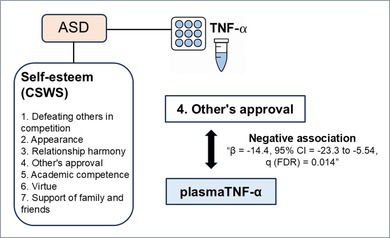

## 1 Introduction

Self‐esteem, a fundamental component of mental well‐being, emerges as a crucial predictor of life satisfaction [1]. Rosenberg's definition (1965) encapsulates self‐esteem as an individual's overall assessment of personal usefulness and worth [2]. High self‐esteem signifies self‐respect and a profound belief in one's inherent value, while low self‐esteem manifests as feelings of worthlessness, often entwined with a perception of fundamental flaws in one's character and accompanied by a perception of fundamental flaws in one's being. In addition, self‐esteem is regarded as an important predictor of mental health, subjective well‐being, social adjustment, and success [3].

Autism spectrum disorder (ASD) stands as a multifaceted neurodevelopmental condition characterized by persistent challenges in social communication, interaction, and the presence of restricted and repetitive patterns of behavior [4, 5, 6, 7, 8]. The inherent difficulties of individuals with ASD in discerning preferences and emotions often result in heightened self‐awareness, contributing to a sense of social isolation and loneliness [9, 10]. Since self‐esteem is lowered by social isolation and loneliness in people living with mental illness [11], the self‐esteem of individuals with ASD is supposed to be lower than that of TD individuals. In fact, several reports indicate that individuals with ASD experience compromised self‐esteem due to their social challenges [12, 13, 14, 15, 16]. However, other existing literature also presents nuanced and controversial results [16, 17, 18]. To shed light on this issue, our study explored the self‐esteem of individuals with ASD using the Contingencies of Self‐Worth (CSW) scale, a comprehensive tool that evaluates self‐esteem across various domains, because of the different domains on which they place value [19, 20, 21]. There are a number of CSW domains, and the measures have been developed and utilized. For example, Crocker et al. developed the measures to evaluate external and internal domains of self‐esteem [20] and other CSW domains have also been developed, such as workplace performance contingent self‐esteem or friendship contingent self‐esteem [22, 23]. Uchida developed and validated the Japanese version of the CSW scale [24].

Understanding the underlying biological basis of the influence of self‐esteem, only a few studies have been reported. O'Donnell et al. and Perez‐Tejada et al. [25, 26] have illuminated associations between immune responses and self‐esteem, particularly tumor necrotic factor‐alpha (TNF‐α) expression, which is a pro‐inflammatory cytokine playing many roles in inflammatory and autoimmune diseases [27]. In the context of ASD, there have been no studies regarding the relationships between self‐esteem and TNF‐α expression; however, TNF‐α emerges as a pivotal molecule in the pathogenesis of ASD, evidenced by its consistently elevated expression [27, 28, 29]. Results from recent meta‐analysis also support these findings, with elevations in TNF‐α [30]. We also reported that TNF‐α mRNA expression in peripheral blood mononuclear cells (PBMCs) and monocyte‐derived macrophages of ASD individuals was significantly associated with their ASD symptoms [31, 32].

Therefore, in this study, we aimed to explore the potential link between self‐esteem and TNF‐α expression in individuals with ASD.

## 2 Method

### 2.1 Participants

High‐functioning adults with ASD were recruited from the outpatient service of the Department of Psychiatry, Nara Medical University Hospital between December 2016 and August 2020. These individuals were diagnosed by two trained psychiatrists according to the criteria of the DSM‐5 (American Psychiatric Association, 2013) and the Japanese version of the Autism Diagnostic Observation Schedule, 2nd edition (ADOS‐2), which is one of the most reliable instruments for diagnosing ASD [33]. None of the TD individuals had a history of psychiatric, neurological, or developmental disorders, as confirmed via the Mini‐International Neuropsychiatric Interview [34]. The TD individuals were also confirmed not to exhibit ASD symptoms using the Autism Spectrum Quotient‐Japanese version (AQ‐J); only those who scored ≤ 25 on this scale were enrolled in this study [35, 36]. To avoid the effects of intellectual disability, individuals with an estimated full‐scale intelligence quotient (FIQ) of less than 70 were excluded from both the ASD and TD groups.

### 2.2 Psychological Test

All the psychological examinations were conducted by an experienced psychologist. The Mini‐International Neuropsychiatric Interview (M.I.N.I.) was used to screen for psychiatric disorders [34]. To assess the core symptoms of ASD, we performed ADOS‐2 [33] and used a self‐administered questionnaire called the Japanese version of the Autism Quotient (AQ‐J) [35, 36]. The FIQ was estimated using the “similarities” and “symbol search” subsets of the Wechsler Adult Intelligence Scale, third or fourth edition [37]. Self‐esteem was measured using the CSW Scale, which was originally developed by Crocker and Wolfe [21]. The Japanese version of the CSW Scale was created by Uchida [24], a self‐report scale containing 35 items. CSW scale measures seven contingencies of self‐esteem: defeating others in competition, appearance, relationship harmony, other's approval, academic competence, virtue, and support of family and friends. Generalized sense of power, that is, the sense of power across the salient relationships, was assessed using the Personal Sense of Power Scale [38]. Participants rated eight items, such as “I can get him/her/them to listen to what I say” and “My wished do not carry much weight.” Participants answered the items related to others in their family and society around them. The Child Abuse and Trauma Scale (CATS) was used to assess the degree of adverse childhood experiences (ACEs) [39]. The CATS questionnaire consists of 38 self‐rated items and retrospectively assesses the frequency of adverse event experiences in childhood and adolescence in five categories: sexual abuse, physical abuse, emotional abuse, neglect, and others.

### 2.3 TNF‐α Levels Measurement

Human whole blood samples were obtained by venipuncture and centrifuged at 1000 *g* for 10 min at room temperature. The supernatant plasma was collected and stored at −80°C until use in ELISA. Quantification of TNF‐α in the plasma of ASD and TD individuals was performed using the Human TNF‐alpha Quantikine HS ELISA kit (HSTA00E) according to the attached protocol (R&D Systems).

### 2.4 Ethics Statement

This study was approved by the appropriate ethics committees of Nara Medical University (No. 1319). This study was conducted in accordance with the Ethics Code of the World Medical Association (Declaration of Helsinki) for experiments involving humans. All participants were given a complete description of the study and provided written informed consent prior to enrollment.

### 2.5 Statistical Analysis

Differences were examined in the demographic characteristics between ASD and TD individuals via the Mann–Whitney test. The chi‐square test was performed to examine differences in sex distribution. Spearman's rank correlation coefficient was used to examine the associations between the levels of TNF‐α expressions and psychological variables. Multiple regression analysis was performed to verify the factors related to CSW scale scores among individuals with ASD. Multiple comparisons were corrected using the Benjamini‐Hochberg procedure to control the false discovery rate (FDR). An FDR threshold of *q* < 0.05 was considered statistically significant. The significance level was set at 0.05. All statistical analyses were performed using SPSS version 29.0.1.0.

## 3 Results

### 3.1 Demographic Characteristics and TNF‐α Expression Levels

A total of 51 high‐functioning individuals with ASD and 34 TD individuals participated in the current study. The age distribution was as follows: TD, 28.0 ± 5.8; ASD, 28.6 ± 7.7. Demographic details are presented in Table 1. There were no significant differences in the age and full‐scale IQ between ASD and TD individuals (*p* > 0.05). Individuals with ASD displayed significantly fewer years of education (*p* < 0.01) and the male percentage was higher in ASD individuals than in TD individuals (*p* < 0.001). Individuals with ASD exhibited significantly higher AQ‐J scores than TD individuals (*p* < 0.0001). There was no significant difference in plasma TNF‐α levels between the ASD and TD individuals (Figure S1).

**TABLE 1:** Demographic characteristics.

	TD	ASD	*p*
	(*n* = 34)	(*n* = 51)	
Age: mean (SD)	28.0 (5.8)	28.6 (7.7)	0.95
Sex: male, *n* (%)	18 (52.9)	41 (80.4)	< 0.001***
Education period: median (IQR)	16.0 (15.5–17.0)	14.0 (12.0–16.0)	0.0012**
Full‐scale IQ: mean (SD)	102.6 (9.8)	102.7 (15.0)	0.62
AQ‐J score: mean (SD)	15.1 (5.4)	32.6 (6.6)	< 0.0001***
ADOS‐2 score: mean (SD)	N/A	15.0 (2.8)	N/A
SA: mean (SD)	N/A	13.3 (2.4)	N/A
RRB: mean (SD)	N/A	1.7 (1.3)	N/A

Abbreviations: ADOS‐2, Autism Diagnostic Observation Schedule–Second Edition; AQ‐J, Autism‐Spectrum Quotient Japanease version; ASD, autism spectrum disorder; IQ, intelligence quotient; IQR, interquartile range; RRB, restricted and repetitive behaviors; SA, social affect; SD, standard deviation; TD, typically developing.

** *p* < 0.01.

*** *p* < 0.001.

### 3.2 Negative Correlation of the External Contingencies of Self‐Worth With TNF‐α Expression in ASD Individuals, Not in TD Individuals

The correlations between CSW and TNF‐α expression were analyzed using three models: crude model, adjusted model for age and sex (Model 1), and adjusted model for age, sex, educational status, FIQ, and AQ‐J (Model 2). After applying FDR correction, both uncorrected raw *p*‐values and FDR‐adjusted *q*‐values are reported in Table 2. Among the three external domains of the seven CSW subscales, “Others' Approval” was significantly negatively correlated with TNF‐α expression in individuals with ASD, whereas “Appearance” and “Relationship Harmony” showed a tendency toward negative correlations with TNF‐α expression in individuals with ASD: Others' Approval (Crude model: *β* = −14.0, 95% CI = −22.9 to −5.10, *p* = 0.003, *q* = 0.021; Model 1: *β* = −14.8, 95% CI = −23.4 to −6.16, *p* = 0.001, *q* = 0.007; Model 2: *β* = −14.4, 95% CI = −23.3 to −5.54, *p* = 0.002, *q* = 0.014). Appearance (Crude model: *β* = −9.47, 95% CI = −18.1 to −0.81, *p* = 0.033, *q* = 0.116; Model 1: *β* = −10.0, 95% CI = −18.8 to −1.20, *p* = 0.027, *q* = 0.063; Model 2: *β* = −10.1, 95% CI = −19.1 to −1.22, *p* = 0.027, *q* = 0.063). Relationship Harmony (Crude model: *β* = −9.88, 95% CI = −19.2 to −0.55, *p* = 0.038, *q* = 0.089; Model 1: *β* = −10.8, 95% CI = −20.0 to −1.61, *p* = 0.022, *q* = 0.077; Model 2: *β* = −10.7, 95% CI = −19.7 to −1.67, *p* = 0.021, *q* = 0.074). The other four subscales: Defeating others in competition, Academic competence, Virtue, and Support of family and friends were not correlated with TNF‐α expressions in ASD individuals. Moreover, none of the CSW subscale scores correlated with TNF‐α expression levels in TD individuals (Table 2). Next, we conducted an analysis including the variable related to childhood adverse experiences—the CATS—as an independent variable. The results showed that TNF‐α expression levels were negatively associated with the CSW subscale score for Others' Approval, independently of the CATS score (Table S1).

**TABLE 2:** Multiple regression analysis for CSW scores as dependent variables.

	TNF‐α			
	*β*	(95% CI)	Raw *p*‐value	*q*‐value (FDR)
1. Defeating others in competition_TD				
Crude model	2.53	(−10.5 to 15.6)	0.696	0.974
Adjusted model 1^a^	4.61	(−10.2 to 19.4)	0.530	0.742
Adjusted model 2^b^	3.92	(−11.8 to 19.7)	0.613	0.858
1. Defeating others in competition_ASD				
Crude model	−6.84	(−15.2 to 1.54)	0.107	0.187
Adjusted model 1^a^	−6.65	(−15.2 to 1.86)	0.123	0.172
Adjusted model 2^b^	−7.14	(−15.9 to 1.59)	0.106	0.186
2. Appearance_TD				
Crude model	8.43	(−6.10 to 23.0)	0.246	0.861
Adjusted model 1^a^	12.4	(−3.85 to 28.6)	0.130	0.303
Adjusted model 2^b^	11.4	(−4.61 to 27.3)	0.156	0.364
2. Appearance_ASD				
Crude model	−9.47	(−18.1 to 0.81)	0.033*	0.116
Adjusted model 1^a^	−10.0	(−18.8 to −1.20)	0.027*	0.063
Adjusted model 2^b^	−10.1	(−19.1 to −1.22)	0.027*	0.063
3. Relationship harmony_TD				
Crude model	−6.09	(−20.0 to 7.83)	0.380	0.665
Adjusted model 1^a^	−2.21	(−17.7 to 13.3)	0.774	0.903
Adjusted model 2^b^	−3.01	(−19.7 to 13.7)	0.713	0.832
3. Relationship harmony_ASD				
Crude model	−9.88	(−19.2 to −0.55)	0.038*	0.089
Adjusted model 1^a^	−10.8	(−20.0 to −1.61)	0.022*	0.077
Adjusted model 2^b^	−10.7	(−19.7 to −1.67)	0.021*	0.074
4. Other's approval _TD				
Crude model	7.24	(−7.15 to 21.6)	0.313	0.730
Adjusted model 1^a^	16.0	(1.43 to 30.5)	0.032*	0.224
Adjusted model 2^b^	15.4	(0.398 to 30.5)	0.045*	0.315
4. Other's approval _ASD				
Crude model	−14.0	(−22.9 to −5.10)	0.003**	0.021*
Adjusted model 1^a^	−14.8	(−23.4 to −6.16)	0.001**	0.007**
Adjusted model 2^b^	−14.4	(−23.3 to −5.54)	0.002**	0.014*
5. Academic competence_TD				
Crude model	−1.53	(−15.0 to 11.9)	0.819	0.956
Adjusted model 1^a^	4.96	(−9.30 to 19.2)	0.483	0.845
Adjusted model 2^b^	4.69	(−10.3 to 19.7)	0.525	0.919
5. Academic competence_ASD				
Crude model	−3.52	(−12.3 to 5.29)	0.426	0.426
Adjusted model 1^a^	−2.92	(−11.8 to 5.98)	0.512	0.512
Adjusted model 2^b^	−3.05	(−12.3 to 6.18)	0.509	0.509
6. Virtue _TD				
Crude model	−12.5	(−25.7 to 0.554)	0.060	0.420
Adjusted model 1^a^	−11.8	(−26.7 to 3.17)	0.118	0.413
Adjusted model 2^b^	−13.2	(−28.8 to 2.38)	0.093	0.326
6. Virtue _ASD				
Crude model	−3.93	(−10.8 to 2.90)	0.253	0.295
Adjusted model 1^a^	−4.65	(−11.2 to 1.95)	0.163	0.19
Adjusted model 2^b^	−4.75	(−11.3 to 1.82)	0.152	0.177
7. Support of family and friends _TD				
Crude model	−0.247	(−14.2 to 13.8)	0.972	0.972
Adjusted model 1^a^	0.269	(−15.7 to 16.3)	0.973	0.973
Adjusted model 2^b^	−1.78	(−18.2 to 14.7)	0.826	0.826
7. Support of family and friends _ASD				
Crude model	−4.98	(−11.5 to 1.49)	0.128	0.179
Adjusted model 1^a^	−5.27	(−11.8 to 1.26)	0.111	0.194
Adjusted model 2^b^	−5.20	(−12.0 to 1.57)	0.129	0.181

*Note:* *p*‐values were adjusted for multiple testing using the Benjamini‐Hochberg procedure to control the false discovery rate (FDR).

Abbreviations: CI, confidence interval; *β*, partial regression coefficient.

^a^ Model 1: adjusted for age and sex.

^b^ Model 2: adjusted for educational status, FIQ, and AQ‐J in addition to model 1.

* Raw *p*‐value or *q*‐value < 0.05.

** Raw *p*‐value or *q*‐value < 0.01.

### 3.3 Trend Toward a Positive Correlation of the Personal Sense of Power With TNF‐α Expression in ASD Individuals, Not in TD Individuals

Among the two dimensions of the Personal Sense of Power scale—Family and Society—scores on the Family subscale exhibited a trend toward a positive association with TNF‐α expression levels in individuals with ASD, although the correlations did not remain statistically significant after applying FDR correction (Crude model: *β* = 13.8, 95% CI = 0.589 to 27.1, *p* = 0.041, *q* = 0.082; Model 1: *β* = 14.0, 95% CI = 0.749 to 27.2, *p* = 0.039, *q* = 0.078; Model 2: *β* = 13.4, 95% CI = 0.724 to 26.1, *p* = 0.039, *q* = 0.078). In contrast, Society scores were not correlated with TNF‐α expression in ASD individuals (Crude model: *β* = 8.57, 95% CI = −3.56 to 20.7, *p* = 0.162, *q* = 0.324; Model 1: *β* = 9.42, 95% CI = −2.91 to 21.7, *p* = 0.131, *q* = 0.262; Model 2: *β* = 9.23, 95% CI = −3.19 to 21.6, *p* = 0.141, *q* = 0.282). None of the scores did correlate with TNF‐α expression levels in TD individuals (Table 3).

**TABLE 3:** Multiple regression analysis for the Personal Sense of Power Scale scores.

	TNF‐α			
	*β*	(95% CI)	Raw *p*‐value	*q*‐value (FDR)
Family contexts_TD				
Crude model	6.83	(−12.8 to 26.5)	0.485	0.970
Adjusted model 1^a^	9.59	(−12.7 to 31.9)	0.387	0.774
Adjusted model 2^b^	11.4	(−10.8 to 33.6)	0.302	0.604
Family contexts_ASD				
Crude model	13.8	(0.589 to 27.1)	0.041*	0.082
Adjusted model 1^a^	14.0	(0.749 to 27.2)	0.039*	0.078
Adjusted model 2^b^	13.4	(0.724 to 26.1)	0.039*	0.078
Society contexts_TD				
Crude model	2.91	(−16.0 to 21.8)	0.756	1.512
Adjusted model 1^a^	5.03	(−15.3 to 25.4)	0.617	1.234
Adjusted model 2^b^	6.25	(−15.9 to 28.1)	0.561	1.122
Society contexts_ASD				
Crude model	8.57	(−3.56 to 20.7)	0.162	0.324
Adjusted model 1^a^	9.42	(−2.91 to 21.7)	0.131	0.262
Adjusted model 2^b^	9.23	(−3.19 to 21.6)	0.141	0.282

*Note:* *p*‐values were adjusted for multiple testing using the Benjamini‐Hochberg procedure to control the false discovery rate (FDR).

Abbreviations: CI, confidence interval; *β*, partial regression coefficient.

^a^ Model 1: adjusted for age and sex.

^b^ Model 2: adjusted for educational status, FIQ, and AQ‐J in addition to model 1.

* Raw *p*‐value or *q*‐value < 0.05.

### 3.4 Negative Correlation of the Autistic Symptoms With TNF‐α Expression in ASD Individuals

Of the three scales of ADOS‐2: Social Affect (SA), Repetitive/Restrictive behavior (RRB), and Total scores, the SA and Total scores were negatively correlated with TNF‐α expression levels in ASD individuals (SA: *r* = −0.351, *p* = 0.012; Total: *r* = −0.317, *p* = 0.023), while the RRB scores were not correlated with those levels (*r* = −0.170, *p* = 0.233) (Table 4). ADOS‐2 was not administered to TD individuals.

**TABLE 4:** Correlation analysis between ADOS‐2 scores and TNF‐α expressions.

	TNF‐α		
	*ρ*	(95% CI)	*p*
ADOS‐2 score_ASD			
SA	−0.351	(−0.58 to −0.075)	0.012*
RRB	−0.17	(−0.43 to −0.12)	0.233
Total score	−0.317	(−0.55 to −0.037)	0.023

*Note:* ADOS‐2: autism diagnostic observation schedule–second edition.

Abbreviations: CI, confidence interval; RRB, restricted and repetitive behaviors; SA, social affect; *ρ*, Spearman's rank correlation coefficient.

* *p* < 0.05.

## 4 Discussion

In this study, we aimed to explore the biological underpinnings of self‐esteem in individuals with ASD, with a focus on TNF‐α expression, given that its abnormalities are frequently associated with ASD pathophysiology as previously reported [40, 41, 42], and its known associations with self‐esteem [25, 26]. Our findings demonstrated that TNF‐α expression levels were negatively correlated with CSW and social interaction impairments in ASD individuals. To the best of our knowledge, this is the first study to suggest a potential implication of an inflammatory cytokine, TNF‐α, in the extent of self‐esteem in individuals with ASD. These results point to a potential link between inflammatory pathways and self‐esteem in ASD.

Self‐esteem has been extensively studied as a mediator of mental health, with its importance widely recognized [43, 44]. Elevated self‐esteem is associated with reduced mental health issues in individuals with ASD [15, 45, 46] and is similarly linked to improved well‐being [47, 48]. However, as noted in the introduction, there remains considerable debate regarding the levels of self‐esteem in individuals with ASD. Therefore, in this study, rather than quantifying self‐esteem directly, we sought to explore its components using the CSW framework. Of the seven domains, TNF‐α expression was found to be significantly negatively correlated with others' approval and exhibited a trend toward negative correlations with appearance and relationship harmony. Notably, appearance, relationship harmony, and others' approval are factors that predominantly relate to interpersonal relationships. These findings suggest that ASD individuals with low TNF‐α expression may experience high self‐esteem in social contexts.

Our prior research indicated that TNF‐α expression in lymphoblasts was negatively correlated with social interaction impairment, as measured by the Autism Diagnostic Interview‐Revised (ADI‐R) in ASD individuals [31]. In this study, we extended these findings by demonstrating that TNF‐α expression in plasma was similarly negatively correlated with the severity of social interaction impairments in ASD individuals, as assessed by the ADOS‐2. These results were contrary to our initial expectation of a positive correlation, given that TNF‐α has been regarded as a biomarker for ASD diagnosis [32]. The negative correlations observed between TNF‐α expression and external CSW scores, even after adjusting for the severity of ASD symptoms, suggest that ASD individuals with lower TNF‐α expression may find higher value in interpersonal relationships, regardless of the extent of their ASD symptoms.

Interpersonal stress‐related cytokines, including interleukin‐1β, interleukin‐6, and TNF‐α, have been studied predominantly in TD individuals [49, 50, 51]. However, no studies have specifically investigated the role of these cytokines in ASD individuals. Although abnormalities in the blood levels of interleukin‐1β, interleukin‐6, and TNF‐α have been reported in ASD individuals [40, 41, 42, 52], a recent meta‐analysis revealed no significant differences in their levels [53]. These inconclusive results may stem from the fact that previous studies did not account for the levels of interpersonal stress experienced by each participant. Recent research has highlighted the phenomenon of “camouflaging” in ASD individuals, wherein they adopt strategies to mask their symptoms in social interactions. Cage and Troxell‐Whitman observed that individuals with ASD frequently engage in camouflaging strategies when interacting with colleagues, friends, and healthcare professionals [15]. Camouflaging is associated with low self‐esteem, anxiety, depression, and an increased risk of suicidality in ASD individuals [54, 55]. Notably, female individuals with ASD are more likely to employ social compensatory behaviors, with camouflaging more prominent in females than males [56, 57]. Although no biomarkers have been identified to explain camouflaging behaviors, our findings suggest that TNF‐α may be related, particularly given the predominance of social compensatory behaviors in females and the associations between interpersonal relationships and TNF‐α expression observed in this study.

In social animals, hierarchical rank has profound effects on behavior, including depression‐like behaviors [58]. Studies have shown that social rank influences the expression of inflammatory cytokines, such as IL‐6 and TNF‐α, with lower‐ranking individuals exhibiting higher cytokine levels [59, 60]. This does not align with our finding that the scores of the Personal Sense of Power were positively correlated with plasma TNF‐α expression. The Personal Sense of Power scale measures an individual's perceived power within their social environment, which is closely related to self‐esteem [38]. To feel empowered, individuals must believe in their ability and worth in their social environment, which is influenced by self‐esteem [61]; conversely, self‐esteem shapes their perception of self‐worth in challenging situations [62]. Our study is the first to apply this scale to individuals with ASD, revealing that the Personal Sense of Power tended to be positively correlated with plasma TNF‐α expression in ASD individuals within family settings, but not in broader social contexts or in TD individuals. These findings suggest that ASD individuals may be more influenced by familial power dynamics than societal ones. Given that social experiences for many ASD individuals are often confined to family interactions, the influence of familial power may be more pronounced. In any case, perceiving a sense of power within family settings may be associated with an increase in TNF‐α expression. These findings led us to consider that individuals with ASD who feel a sense of superiority only within their family, but not in society, may not actually experience a high level of self‐esteem, which differs from the findings of studies on TD individuals [38].

In recent years, accumulating evidence has indicated that ACEs, histories of abuse, and disruptions in attachment formation are associated with long‐term health risks, particularly through the dysregulation of immune function. Elevated levels of pro‐inflammatory cytokines, such as TNF‐α and IL‐6, have consistently been observed in individuals exposed to early‐life psychosocial stress [63, 64], as well as in those with insecure or disrupted attachment histories [65]. These findings underscore the importance of considering developmental and psychosocial backgrounds when interpreting individual differences in immune and inflammatory markers, including TNF‐α. In this study, we found that TNF‐α expression levels were negatively correlated with the CSW subscale score for Others' Approval, independently of childhood adversity as measured by the CATS score, suggesting the involvement of additional factors.

While TNF‐α is widely recognized for its pro‐inflammatory properties, more recent studies have highlighted its involvement in a variety of non‐immune biological processes. These include lipid metabolism and neuroendocrine regulation. For instance, TNF‐α has been shown to regulate lipid metabolism by influencing hepatic lipid synthesis, adipocyte lipolysis, and overall energy balance, potentially contributing to metabolic dysregulation [66, 67, 68]. Recent findings have also indicated that lipid metabolism may be directly related to psychological traits such as self‐esteem and resilience [69]. It also modulates neuroendocrine function by interacting with hormonal pathways such as insulin signaling and plays a significant role in the development of insulin resistance [70]. Insulin resistance has been reported to be positively associated with the severity and chronicity of depression [71] and negatively associated with self‐efficacy in patients with type 2 diabetes [72]. These pleiotropic roles of TNF‐α suggest that its impact extends well beyond classical immune responses and may be relevant to psychological and behavioral outcomes such as self‐esteem.

This study had limitations. First, participants were not randomly sampled from the general population and, therefore, may contain some bias. Second, although we statistically adjusted for the sex distribution, a difference existed in our cohort.

In conclusion, while it is commonly believed that individuals with ASD experience diminished self‐worth in interpersonal relationships, our findings indicate that, regardless of the severity of their autistic symptoms, those who derive self‐esteem from such relationships exhibit lower levels of TNF‐α, an inflammatory cytokine. Moreover, those who perceive a sense of power within familial contexts, not within societal settings, exhibit higher levels of TNF‐α, suggesting that the process of developing self‐esteem may differ between ASD and TD individuals. Although the precise role of TNF‐α remains to be fully elucidated, our results highlight the biological significance of how individuals with ASD perceive their interpersonal relationships.

## Author Contributions

Y.K., T.Y., M.M.: writing – review and editing. Y.K., T.Y., T.T., K.O., R.I., K.Y. M.T.: data collection. T.Y., T.O.: data curation, drawing table. Y.K., T.Y.: investigation. Y.K., T.Y.: project administration. N.I., M.M.: manuscript review. T.Y.: drawing figures. T.O., N.I., M.M.: supervision, manuscript editing.

## Ethics Statement

The study was approved by the Nara Medical University Ethics Committee (No. 1319).

## Consent

Written informed consent was obtained from all participants.

## Conflicts of Interest

The authors declare no conflicts of interest.

## Supporting information


**Figure S1:** npr270047‐sup‐0001‐FigureS1.pdf.

**Table S1:** Multiple regression analysis for CSW scores as dependent variables and CATS score added as an independent variable.

## Data Availability

The datasets used in this study are available in Supporting Information.
