# Supplementary figures and images for: Associations of TNF‐α Expression With Self‐Esteem in Autism Spectrum Disorder
Source: Neuropsychopharmacol Rep. 2025 Oct 8;45(4):e70047. doi: 10.1002/npr2.70047 (PMC12508256; doi:10.1002/npr2.70047)

# Supplementary Figure 1

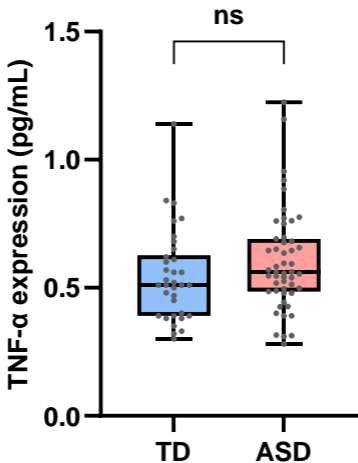

Supplement: Supplementary file 1 — Figure S1: npr270047‐sup‐0001‐FigureS1.pdf. [file NPR2-45-e70047-s002.pdf]
